# White matter differences between younger and older adults revealed by fixel-based analysis

**DOI:** 10.1016/j.nbas.2024.100132

**Published:** 2024-11-23

**Authors:** Feliberto de la Cruz, Andy Schumann, Katrin Rieger, Daniel Güllmar, Jürgen R. Reichenbach, Karl-Jürgen Bär

**Affiliations:** a Lab for Autonomic Neuroscience, Imaging and Cognition (LANIC), Department of Psychosomatic Medicine and Psychotherapy, Jena University Hospital, Jena, Germany; b Medical Physics Group, Institute of Diagnostic and Interventional Radiology, Jena University Hospital, Jena, Germany

**Keywords:** Aging, White matter, Fixel-based analysis, Risk factors

## Abstract

The process of healthy aging involves complex alterations in neural structures, with white matter (WM) changes significantly impacting cognitive and motor functions. Conventional methods such as diffusion tensor imaging provide valuable insights, but their limitations in capturing complex WM geometry advocate for more advanced approaches. In this study involving 120 healthy volunteers, we investigated whole-brain WM differences between young and old individuals using a novel technique called fixel-based analysis (FBA). This approach revealed that older adults exhibited reduced FBA-derived metrics in several WM tracts, with frontal areas particularly affected. Surprisingly, age-related differences in FBA-derived measures showed no significant correlation with risk factors such as alcohol consumption, exercise frequency, or pulse pressure but predicted cognitive performance. These findings emphasize FBA’s potential in characterizing complex WM changes and the link between cognitive abilities and WM alterations in healthy aging. Overall, this study advances our understanding of age-related neurodegeneration, highlighting the importance of comprehensive assessments that integrate advanced neuroimaging techniques, cognitive evaluation, and demographic factors to gain insights into healthy aging.

## Introduction

The healthy aging process is a multifaceted phenomenon involving numerous physiological, psychological, and neurobiological changes [1]. White matter (WM) alterations have received particular attention due to their significant role in cognitive and functional decline [2], [3], [4]. WM, composed primarily of myelinated axons connecting distinct brain regions, forms a complex neural network that enables efficient communication within the central nervous system. With advancing age, individuals experience a series of WM modifications associated with differences in cognitive processing, sensory perception, and motor function [2]. Thus, gaining insight into the complex interplay between aging and WM changes is crucial for understanding cognitive decline and developing interventions to reduce the negative impacts of age-related neurodegeneration.

Recent advancements in neuroimaging techniques and analysis tools have given researchers unprecedented opportunities to investigate the underlying mechanisms driving aging-related WM changes [5], [6]. In particular, diffusion-weighted imaging (DWI) has emerged as a promising imaging technique, providing non-invasive insights into the brain’s WM tracts. DWI measures the mobility of water molecules within biological tissues, which can be affected by factors such as tissue integrity, axonal density, and myelination [7], [8]. However, it is worth noting that most studies on WM changes in aging have predominantly employed diffusion tensor imaging (DTI). DTI-based metrics like fractional anisotropy, radial diffusivity, and axial diffusivity serve as indirect indicators of WM integrity [9], [10]. While DTI performs well in regions characterized by a dominant fiber direction, its efficacy diminishes in regions with complex fiber geometry and various fiber populations [11]. Notably, DTI cannot directly reveal changes in WM influenced by distinct biophysical properties such as axon diameter, density, and myelin concentration [12], [13].

In contrast, a recently developed analytical framework known as fixel-based analysis (FBA) introduces a novel avenue for assessing WM characteristics [14], [15]. This methodology enables the statistical examination of quantitative measures in complex fiber geometry. Within the FBA framework, a “fixel” denotes a distinct fiber population within a voxel. FBA-derived metrics provide insights into variations in fiber density (FD) within a fiber bundle, differences in fiber-bundle cross-sectional area (FC), and differences arising from combined processes (FDC = FD*FC) [14]. FD represents the intra-axonal volume of the fiber population, while FC captures macrostructural, morphological changes in the cross-sectional area of WM fiber bundles, akin to those resulting from axon loss or myelin damage. Consequently, FDC indicates the overall quantity of axons within the fiber tract, accounting for variations in both FD and FC. In summary, FBA emerges as a method offering increased anatomical specificity compared to DTI, consequently enhancing the biological interpretability of findings [16].

To date, only two studies have used FBA to assess WM differences between younger and older adults. In one study by Kelly et al. (2021), age-related differences in fractional anisotropy were linked to fiber population complexity within a voxel. Specifically, they found a strong inverse relationship between fractional anisotropy and crossing fibers in regions exhibiting a significant age effect on fractional anisotropy. This study also showed that age-related differences in FD are most pronounced in specific WM areas, including the fornix, bilateral anterior internal capsule, forceps minor, body of the corpus callosum, and corticospinal tract. Meanwhile, differences in FC were most notable in the cingulum bundle and forceps minor [6]. In another study by Choy et al. [5], WM changes across the adult lifespan were examined using FBA in a cross-sectional design. Their findings also indicated significant and widespread age-related alterations in FD and FC across the entire brain. Interestingly, certain fiber bundles, such as the anterior thalamic radiation, corpus callosum, and superior longitudinal fasciculus, exhibited a significant negative relationship between age and FD but not with FC. It is worth noting that none of these studies explored the relationship between FBA-derived metrics and well-established factors known to influence WM structure, such as alcohol consumption [17], [18], physical activity [19], [20], or pulse pressure [21], [22].

Those risk factors play a critical role in understanding the variability in WM integrity across individuals [17], [18], [19], [20], [21], [22]. Alcohol consumption has been shown to cause neurotoxic effects on WM, particularly in regions such as the corpus callosum and corona radiata, potentially accelerating the age-related decline in cognitive functions [17], [18]. Similarly, pulse pressure, an indicator of arterial health, has been associated with WM damage [21], [22]. Elevated pulse pressure has been linked to increased WM hyperintensities and microstructural damage, particularly in regions like the corpus callosum and the periventricular WM, which could worsen the age-related decline in WM integrity. On the other hand, physical activity has a protective effect on WM structure, with studies showing that higher levels of fitness are associated with increased WM integrity [19], [20]. Chen et al. [19] reported that long-term exercise delays the age-related decline in spatial learning ability and WM atrophy by protecting against age-related changes in myelinated fibers and oligodendrocytes in the WM. Incorporating these risk factors when assessing age-related WM differences between individuals may help to better explain the observed variability in WM integrity.

In this study, we used DWI data from a cohort of 120 healthy volunteers, divided into 60 younger and 60 older adults. We aimed to characterize age-related WM differences across the entire brain using FBA. Consistent with previous reports [5], [6], we hypothesized widespread differences between younger and older individuals. To gain a better understanding of the specific WM pathways affected in older subjects, we classified significant fixels into 11 canonical WM tracts using established anatomical atlases. Additionally, we complemented our study by conducting linear regression analyses to explore potential risk factors contributing to WM differences, such as pulse pressure, alcohol consumption, and frequency of physical activities. Furthermore, we examined the relationship between the degree of WM differences in older individuals and their cognitive performance. Given the variable associations between FBA metrics and age—both positive and negative [5]—we anticipated a diverse pattern of correlations between FBA metrics in specific tracts and risk factors or cognitive performance.

## Materials and methods

### Participants

The neuroimaging data used in this study are part of a larger ongoing investigation into healthy aging, where we had initially collected only physiological data [23]. Recruitment efforts involved advertising at social clubs, the University of Jena, and surrounding areas, spanning a recruitment period of approximately two years. Inclusion criteria were: age over 18 years, absence of diagnosed neurological or psychiatric disorders, fluency in the German language, willingness to participate in and comply with study procedures and a normal electrocardiogram. Exclusion criteria included: presence of medical conditions or medications affecting brain structure or function (e.g., stroke, traumatic brain injury, substance abuse or dependence), and MRI contraindications (e.g., metallic implants, claustrophobia). Informed written consent was obtained from all participants 24 h in advance. The study was conducted in accordance with protocols approved by the local Ethics Committee of the University Hospital Jena and adhered to the guidelines outlined in the Helsinki Declaration (2013).

We acquired neuroimaging data from 120 participants, and surprisingly, no participants in the range of 38 and 49 years old took part in the study. Specifically, there were 60 participants aged below 38 years old (55 % women; mean age: 23.5 ± 3.2 yrs) and 60 participants aged above 49 years old (60 % women; mean age: 67.1 ± 7.3 yrs), which conveniently facilitated the definition of age thresholds for grouping. Three younger and four older participants were left-handed, while the remaining were right-handed, as determined by the modified version of Annett’s handedness inventory [24]. No participant was excluded due to poor neuroimaging data quality. All demographic and clinic characteristics are listed in Table 1.

**Table 1:** Demographics and clinical characteristics.

General	Younger adults n = 60	Older adults n = 60	p-value
Age [y]	23.5 ± 3.2 (18 to 37)	67.1 ± 7.3 (50 to 85)	*<0.001*†
Female	33	36	*0.712*‡
Male	27	24	
TIV [mm^3^]	1601974 ± 172444	1561377 ± 158785	*0.182*†
TMT-A	24.4 ± 7.5 (8.4 to 41.9)	36 ± 11.8 (14.6 to 80)	*<0.001*†
TMT-B	49.7 ± 15.8 (23.4 to 86)	81.2 ± 29.3 (36.7 to 189.1)	*<0.001*†
PP [mm Hg]	45.8 ± 10.9 (28 to 67)	48.3 ± 14.3 (27 to 110)	*0.507*†

*Frequency alcohol/week*^a^			
0	7	12	*0.348*‡
1	32	24	
2	15	15	
3	0	0	
>4	3	7	

Glasses of alcohol/day*			
0	8	13	*0.040*‡
1	0	1	
2	32	39	
3	0	0	
>4	20	7	

*Physical activity (hour/week)*^b^			
0	7	14	*0.030*‡
1	15	24	
2	0	0	
3	20	15	
>4	15	6	

*Non-neurological disorders*			
Hypertension	1	20	
High cholesterol	0	1	
Diabetes	0	1	
Others	8^c^	14^d^	

*Medication for:*			
Hypertension	0	17	
High cholesterol	0	1	
Others	12	12	

Values for continuous variables are presented as mean ± SD (range).

Values for categorical and discrete variables are presented as number of participants.

*A glass of alcohol refers to a serving of any alcoholic beverage, regardless of the quantity.

TIV = total intracranial volume, TMT = trail making test, PP = pulse pressure.

† *t*-test.

‡ χ2 test.

a 3 younger and 2 older participants refused to answer.

b 2 younger and 1 older participants refused to answer.

c 2 neurodermitis, 1 psoriatic arthritis, 1 Hashimoto’s disease, 1 hypothyroidism, 1 celiac disease, 1 polycystic ovary syndrome, 1 rheumatoid arthritis.

d 3 Osteoporosis, 2 cancer history, 2 arthrosis, 1 rheuma, 1 bronchitis, 1 systematic lupus erythematosus, 1 hypothyroidism, 1 blepharitis, 1 chronic lymphocytic leukemia, 1 myeloproliferative neoplasms.

### Risk factors and cognitive assessment

As individuals age, their cardiovascular system undergoes significant changes, including increased arterial stiffness, leading to a rise in pulse pressure (PP) [25]. To calculate PP, we collected continuous non-invasive blood pressure data over a 20-minute period with participants in a supine position, utilizing a CNAP device (CNSystems Medizintechnik AG, Graz, AT) in the laboratory. The first 5 min of data were excluded to eliminate potential artifacts. Systolic and diastolic blood pressure values were automatically detected using algorithms provided by the device, and PP was computed as the difference between systolic and diastolic blood pressure.

In addition to cardiovascular measurements, participants completed a self-reported questionnaire assessing other relevant risk factors in this study. Specifically, they reported the frequency of physical activity and alcohol consumption per week, as well as the number of glasses of alcohol consumed per day. These categorical variables were scaled from 0 to 4, as shown in Table 1.

Cognitive performance was evaluated using the neuropsychological Trail Making Test (TMT) [26]. The TMT test comprises two parts: in Part A, participants draw lines sequentially connecting 25 encircled numbers distributed on a sheet of paper, while in Part B, they also draw lines to connect the circles in an ascending pattern, but with the added task of alternating between numbers and letters. Hence, TMT-A evaluates psychomotor speed and complex visual attention, while TMT-B evaluates mental flexibility and executive functioning. Each part’s score represents the amount of time required to complete the task.

### Scanning protocol

MRI data were collected using a 3T whole-body MRI system equipped with a 64-channel head matrix coil (MAGNETOM Prisma Fit, Siemens Healthcare, Erlangen, Germany). Our DWI sequence used a multiband, multi-shell acquisition protocol with the following parameters: repetition time/echo time = 3318/87 ms, voxel size = (1.5x1.5x1.5) mm^3^, 96 axial slices, and a multiband acceleration factor = 4. We used three diffusion-weighted shells: b = 800 s/mm^2^ (16 volumes), b = 1600 s/mm^2^ (32 volumes), and b = 2500 s/mm^2^ (48 volumes), along with 8 non-diffusion-weighted volumes (b = 0 s/mm^2^) [27]. The total acquisition time for the DWI was approximately 6 min. To correct for geometric distortions induced by strong susceptibility differences in the human head, we acquired a second DWI scan with the same parameters but with opposing phase-encoding direction. After DWI, we acquired a high-resolution anatomical T1-weighted volume scan using a magnetization-prepared rapid gradient echo (MP-RAGE) sequence with parameters: repetition time/echo time = 2400/2.3 ms, inversion time = 1040 ms, flip angle = 8°, 320 axial slices, and voxel size = (0.7 x 0.7 x 0.7) mm^3^. We used the MP-RAGE image to estimate total intracranial volume with FreeSurfer v7.3.2 [28],and included total intracranial volume and sex as covariates in subsequent statistical analyses.

### Image processing

We utilized the multi-shell multi-tissue FBA pipeline (https://mrtrix.readthedocs.io/en/latest/fixel_based_analysis/mt_fiber_density_cross-section.html), which incorporates routines from MRtrix3 v3.0.3 [29], FSL v6.0.5 [30], and ANTs v2.3.5 [31] software for the preprocessing and analysis of DWI data. Briefly, the DWI data underwent denoising and correction for susceptibility-driven distortions, eddy currents, and bias fields. Subsequently, we derived the average response functions across all subjects for WM, gray matter, and cerebrospinal fluid [32]. These average response functions are required for the multi-shell multi-tissue constrained spherical deconvolution algorithm, which enables the computation of continuous fiber orientation distribution (FOD) at each voxel [33]. In order to make FOD absolute amplitudes comparable between subjects, we normalized the resulting FOD map using multi-tissue informed intensity normalization in the log-domain. To achieve spatial alignment between subjects, we created a study-specific unbiased FOD template, employing FOD images from 40 subjects (20 younger and 20 older adults) through an iterative co-registration approach [34]. Afterwards each subject’s FOD image was non-linearly aligned with the study template, followed by segmenting FOD lobes to identify the number and orientation of fixels in each voxel. Finally, we calculated three FBA-based metrics: FD, log(FC), and FDC across all WM fixels. We utilized log(FC) instead of FC to ensure that the data were centered around zero and normally distributed.

### Statistical analysis

#### Whole-brain FBA

For statistical comparison of whole-brain differences in FBA-based metrics between younger and older adults, we applied a fixel-wise general linear model controlled by total intracranial volume and sex. This analysis combined connectivity-based fixel enhancement (CFE) and non-parametric permutation testing [15]. The CFE method is a threshold-free cluster enhancement approach that uses probabilistic tractography to identify structurally connected fixels that are likely to share underlying anatomy. To this end, we generated a whole-brain tractogram of 20 million streamlines using a probabilistic algorithm on the population FOD template. Subsequently, we reduced the tractogram to 2 million streamlines using the spherical-deconvolution informed filtering of tractograms algorithm to mitigate reconstruction biases [35]. We performed ten thousand permutations to compute a null distribution of maximum cluster-enhanced test statistics across fixels. The true test statistic for each fixel was then compared against this null distribution to determine the final p-value of the test. A family-wise error (FWE) corrected p-value was assigned to each fixel, and those with *p*_FWE_ < 0.05 were deemed statistically significant.

#### Association of FBA metrics with risk factors at specific tracts

**Fig. 1:**
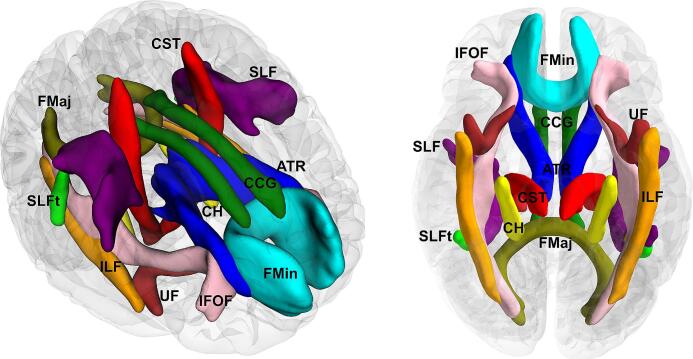
The 11 canonical tracts utilized for fixel assignments in FBA. ATR, anterior thalamic radiation; CCG, cingulum (cingulate gyrus); CH, cingulum (hippocampus); CST, corticospinal tract; FMaj, forceps major; FMin, forceps minor; IFOF, inferior fronto-occipital fasciculus; ILF, inferior longitudinal fasciculus; SLF, superior longitudinal fasciculus; SLFt, superior longitudinal fasciculus (temporal); UF, uncinate fasciculus.

To further analyze our FBA findings, we associated significant fixels with canonical major WM tracts. To accomplish this, we utilized the John Hopkins University (JHU) WM atlas, integrated with FSL, to create masks for 11 major tracts (Fig. 1). In the JHU WM atlas, tracts with bilateral pairs include the anterior thalamic radiation, cingulum cingulate gyrus, cingulum hippocampus, corticospinal tract, inferior fronto-occipital fasciculus, inferior longitudinal fasciculus, superior longitudinal fasciculus, superior longitudinal temporal fasciculus, and uncinate fasciculus, while forcep minor and forcep major are single tracts extending across both hemispheres.

Since the JHU WM atlas is in MNI space, we first aligned an MNI T1 template (also part of FSL) to the population FOD template using the ANTs toolkit [36]and applied the transformation parameters to the JHU WM atlas. Masks for each tract were generated, and we calculated the mean FD, FC, and FDC values for all significant fixels from the group comparison within each mask. Tracts in the left and right hemispheres were analyzed separately to account for potential hemispheric asymmetries. For each average FBA metric (dependent variable), we performed a linear regression model with risk factors and cognitive performance (TMT-A/B) as independent variables, controlling for sex and total intracranial volume. In total, we conducted 260 post-hoc statistical tests, and the associated p-values were corrected for multiple comparisons using False Discovery Rate (FDR) correction. An FDR corrected p < 0.05 was considered statistically significant.

## Results

### Demographic and clinical characteristics

The demographic and clinical characteristics of each group are summarized in Table 1. There were no significant differences between the younger and older adult groups at a significance level of p < 0.05 in terms of sex (Χ^2^ = 0.136, p = 0.712), total intracranial volume (t = 1.34, p = 0.182), pulse pressure (t = 0.667, p = 0.507), and frequency of alcohol consumption per week (Χ^2^ = 4.45, p = 0.348). However, significant differences were observed between the groups in TMT-A (t = 6.39, p < 0.001), TMT-B (t = 7.23, p < 0.001), glasses of alcohol consumed per day (Χ^2^ = 8.63, p = 0.040), and the number of hours of physical activity per week (Χ^2^ = 8.95, p = 0.030), along with the grouping variable age (t = 42.18, p < 0.001).

### Whole-brain FBA

**Fig. 2:**
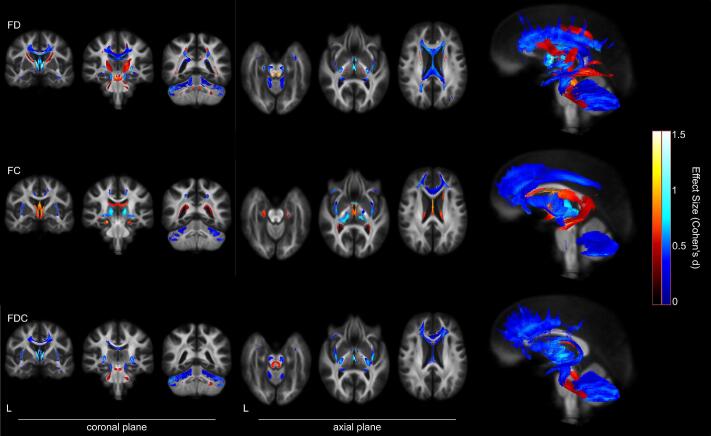
WM areas with significantly lower and greater fiber density (top row), fiber bundle cross-section (middle row), and the combined measure of FDC (bottom row) in older compared with younger adults, displayed on the WM population template. Blue colors represent fixels where the corresponding measure was significantly lower in the older group, while red-yellow colors represent fixels where the corresponding measure was significantly greater in the older group. In both color maps, brighter colors indicate a larger effect size (Cohen’s d). The first three images from the left side are displayed in the coronal plane, and the subsequent images are presented in the axial plane. The sagittal plane on the right side shows all fixels that met the statistical threshold of *p*_FWE_ < 0.05. (For interpretation of the references to color in this figure legend, the reader is referred to the web version of this article.)

The whole-brain FBA comparing younger and older adult participants revealed substantial WM differences at p_FWE_ < 0.05. These differences mainly encompassed reduced FD, FC and FDC in older participants, as depicted in Fig. 2. Specifically, reductions in FD were evident across multiple fixels within the anterior thalamic radiation, corticospinal tract, body of the corpus callosum, forceps minor, fornix and middle cerebellar peduncle. Additionally, reductions in FC were predominantly observed within the anterior thalamic radiation, corticospinal tract, hippocampus cingulum, forceps minor, middle cerebellar peduncle, and external capsule. FDC in older adults was significantly lower in the anterior thalamic radiation, corticospinal tract, body of the callosum, forceps minor, fornix, middle cerebellar peduncle, and inferior fronto-occipital fasciculus. Moreover, it is worth noting that overall age-related effects were more prominent for all FBA metrics in the frontal than in the posterior brain regions.

**Fig. 3:**
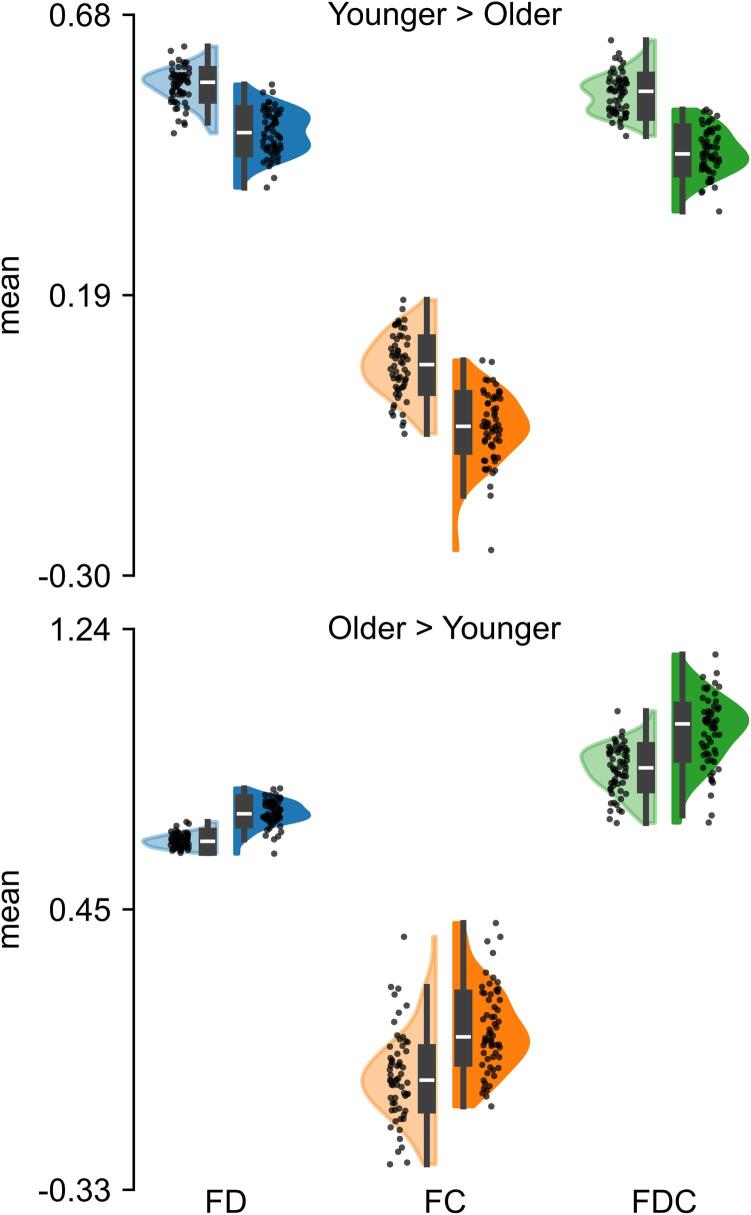
Violin plots of FBA metrics sampled from significant fixels comparing younger (bright colors) and older (dark colors) participants. The top plot shows the contrast younger > older, and the bottom plot shows the contrast older > younger. FD = fiber density, FC = fiber bundle cross-section, and FDC = FD*FC.

Importantly, though to a lesser extent, the opposite contrast also identified a set of WM regions where FBA metrics were greater in older participants than their younger counterparts (see Fig. 2). These group differences were driven by greater FD and included fixels within the bilateral anterior thalamic radiation, superior portion of bilateral corticospinal tracts, and superior cerebellar peduncles. Greater FC was primarily localized in fixels of the middle portion of the left corticospinal tract, the body of the corpus callosum and bilateral cingulum hippocampus. Lastly, FDC in older adults exhibited significantly greater differences in small portions of the middle and superior cerebellar peduncles and bilateral fornix. Fig. 3 summarizes the group comparisons of fixel metrics, illustrating the magnitude of the observed differences in FBA metrics between younger and older participants.

### Association of FBA-metrics with risk factors

**Fig. 4:**
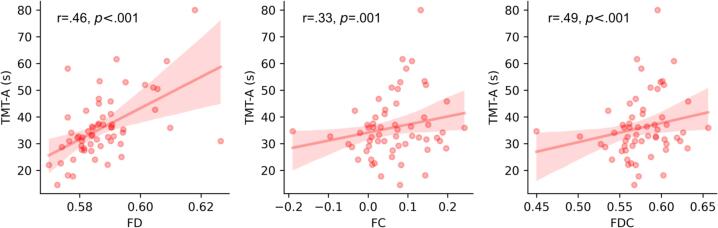
Illustrates a representative correlation between fiber density (FD), fiber bundle cross-section (FC), and the combined measure of fiber density and cross-section (FDC) in the right anterior thalamic radiation with TMT-A in older participants. Each data point represents the mean FD, FC, and FDC extracted from significant fixels within the right anterior thalamic radiation. For clarity, the Pearson correlation coefficient and the corresponding p-value between TMT and FBA metrics are presented, rather than the pseudo R-squared of the linear model’s goodness-of-fit (see Table 2 for the results of the linear model).

We further explored whether the degree of WM differences in older participants could be related to risk factors such as alcohol consumption, pulse pressure, and less physical activity as well as their cognitive performance (TMT-A and TMT-B), controlled by sex and total intracranial volume. We did not find any significant association between differences in FBA metrics in older participants and the examined risk factors. However, we did observe significant associations between greater FBA metrics and cognitive performance in several major tracts, as depicted in Fig. 4 for the right anterior thalamic radiation. Notably, the relationship between FBA and TMT was mainly influenced by the relationship between FD and TMT-A. In total, 12 out of 260 linear models assessing FBA and TMT survived FDR correction and are illustrated in Table 2.

**Table 2:** Linear models surviving FDR correction. The models assessed the association between FBA metrics (dependent variable) and TMT (independent variable), controlling for sex and total intracranial volume.

FBA (DV)	TMT (IV)	tract	Left/Right	# fixels	Pseudo-R^2^	FDR p-value
FD	TMT-A	ATR	L	511	0.201	0.015
FD	TMT-A	ATR	R	408	0.234	0.006
FD	TMT-A	IFOF	L	120	0.351	<0.001
FD	TMT-A	IFOF	R	199	0.269	0.007
FD	TMT-B	IFOF	L	120	0.178	0.035
FD	TMT-A	ILF	L	55	0.303	< 0.001
FD	TMT-A	ILF	R	100	0.218	0.015
FD	TMT-B	ILF	L	55	0.178	0.025
FD	TMT-A	SLF	R	87	0.219	0.010
FC	TMT-A	ATR	R	112	0.742	0.015
FDC	TMT-A	ATR	L	79	0.548	0.026
FDC	TMT-A	ATR	R	37	0.677	<0.001

FBA = fixel based analysis, DV = dependent variable, TMT = trail making test, ID = independent variable, FD = fiber density, FC = fiber-bundle cross-section, FDC = FD*FC, ATR = anterior thalamic radiation, IFOF = inferior fronto-occipital fasciculus, ILF = inferior longitudinal fasciculus, SLF = superior longitudinal fasciculus.

## Discussion

Our whole-brain FBA comparing younger and older adult participants revealed significant WM alterations. These differences were characterized by substantial reductions in all FBA metrics across multiple fixels within key WM tracts, including the anterior thalamic radiation, corticospinal tract, body of the corpus callosum, and forceps minor in the older cohort. Although to a lesser extent, older adults also exhibited increases in all FBA metrics in some major tracts, primarily driven by increased FD. Notably, we did not find significant associations between differences in FBA metrics in older adults and risk factors such as alcohol consumption, pulse pressure, and frequency of physical activity per week. However, in several major tracts, we observed strong correlations between higher FBA metrics in the older group and cognitive performance, specifically between FD and TMT-A. These findings indicate that while there are notable WM alterations in older adults, these differences are not significantly linked to common risk factors but show a strong connection with cognitive performance in specific brain regions.

Our main results of selective degeneration along specific fiber pathways in older adults align with earlier and recent studies of WM deterioration in healthy aging. FD reductions in forcep minor, corticospinal tracts, and corpus callosum may underlie cognitive dysfunctions in older individuals [37]. These pathways connect brain regions previously implicated in healthy aging, suggesting a structural basis for functional network-based alterations. For example, disconnections within the corticospinal tract have been associated with functional disruptions between sensorimotor regions [38]. In addition, a large body of literature suggests that most age-related declines in motor performance may be related to the reduction in the fiber number of the corticospinal tract [38], [39], [40], [41]. Likewise, the structural disruption of the anterior thalamic radiation corresponds to functional disconnections of the anterior and ventromedial nuclei of the thalamus to the prefrontal cortex, including anterior cingulate and dorsolateral frontal regions [42], [43]. These brain regions are central to the attention-control network and play a crucial role in human cognition [44], [45]. Affected fiber pathways connecting the anterior and ventromedial nuclei of thalamus to the prefrontal cortex may impair the information flow across these brain regions, leading to a deficit in executive function skills [46], a domain commonly affected in older individuals [47], [48]. Similarly, the forceps minor, the anterior part of the corpus callosum, connects the homologous regions of the anterior frontal lobes between two hemispheres [49], [50]. Among the regions connected, the frontopolar cortex is essential for complex cognitive behaviors and decision-making in human and non-human primates [51], [52].

One intriguing aspect of our study is that alterations in FD predominantly influenced the differences in FBA metrics among older adults. This observation aligns with previous research indicating relatively preserved fiber cross-sections in several white matter fiber tracts with age [5]. There are a few potential explanations for this phenomenon. One possibility is an increase in the extra-axonal space due to the degeneration of fibers, leading to a reduction in FD. Another explanation could be the filling of the extra-axonal space with other extracellular materials, regardless of axonal loss. Moreover, histological evidence suggests that aging-related axonal loss primarily occurs in smaller-diameter axons [53], [54]. This implies that FC in regions containing axons of different calibers like the corpus callosum [55], [56] are less vulnerable to the aging process.

Our analysis revealed a gradient in FBA metrics along the anterior-posterior axis, indicating more significant age effects in anterior regions. This finding agrees with the research conducted by Kelley et al. [6] and supports the overall concept of varied vulnerability across different brain regions [57], [58]. A recent hypothesis regarding brain aging suggests that this varying vulnerability in regions might be linked to the different developmental timelines of brain areas. Specifically, it posits that WM fiber that myelinate later possess thinner myelin sheaths compared to those tracts that myelinate earlier in development, making them more susceptible to age-related changes [59]. Consistent with this spatial heterogeneity in WM changes, we observed several regions in which older adults had greater FBA metrics than younger participants. Although these increases were less prominent than the reductions in FBA metrics, this observation highlights the multifaceted nature of WM aging. For instance, a greater FC may indicate populations of fibers with larger diameters, either due to a greater size of the axon bundle or a higher degree of myelination [14]. Moreover, partially in line with other recent FBA studies [5], [6], FBA metrics were greater in older adults in portions of the superior corticospinal tract, fornix, cingulum hippocampus and body of the corpus callosum. In particular, our result of increased FC in the cingulum hippocampus is consistent with a report assessing WM morphology across the adult lifespan [5].

The multiple linear regression model indicated that the magnitude of FBA metrics in significant fixels was not associated to risk factors in older adults, such as pulse pressure, alcohol consumption and frequency of physical activities per week. The lack of significant associations might be attributed to the nature of our variables. Alcohol consumption and hours of physical exercise per week were categorical, self-report, and therefore subject to participant response bias. Pulse pressure, defined as the difference between systolic and diastolic pressures, is an index of arterial wall stiffness, which changes with age. A recent study utilizing a large database (UK Biobank) demonstrated a weak correlation between pulse pressure and WM integrity, assessed by fractional anisotropy, in the corpus callosum [21]. However, this association was limited to individuals within the age range of 45–75 years. It is important to note that FBA and tensor-based metrics might reflect different underlying biological mechanisms, potentially leading to different results.

In general, using FBA has advantages over diffusion tensor methods. FBA’s fiber tract-specific model provides more directly understandable measures of altered structural connectivity compared to tensor metrics like fractional anisotropy. FBA is especially helpful in reducing misinterpretations in brain regions with complex crossing fiber architecture, which is present in around 90 % of brain voxels [11]. Recent studies have further confirmed these advantages by showing that voxel-averaged tensor-based metrics can inaccurately indicate unchanged or increased values in regions with complex fiber arrangements, where a decrease is expected [12], [13].

Our study has limitations that warrant acknowledgment. Although our data had sufficient angular resolution (104 gradient directions; 45 is the minimum required to fully characterize the DW signal [60] and employed three shells, it’s important to recognize that the data acquisition was not fully optimized for FBA. Notably, the highest b-value utilized in our study was 2500 s/mm^2^. A higher b-value could potentially enhance sensitivity to diffusion within the intra-axonal compartment, thereby reducing the impact of extra-axonal diffusion contamination on fibre density [61], [62]. For instance, Genc et al. [61] demonstrated that b-values higher than 4000 s/mm^2^ are necessary to achieve a high correspondence between fiber density and the underlying intra-axonal fiber properties [61]. Additionally, our study examined WM differences regardless of sex differences. Recent advanced DWI studies suggest that age-related brain changes might commence earlier in males than in females [63]. Due to our limited sample size, we opted against conducting a sex-based analysis as it might not yield conclusive results. The lack of association between risk factors and FBA metrics should be interpreted with caution. For example, more than 25 % of the older participants reported using anti-hypertensive medication, which might have impacted the range of pulse pressure values and obscured any potential correlation with FBA metrics. Another limitation is the use of the JHU atlas to label significant fixels into 11 WM tracts. This approach may have introduced some inaccuracies, as only 11 tracts may not be sufficient to differentiate distinct WM pathways. Fixels labeled as part of the same tract could, in fact, belong to different tracts. Ideally, WM tracts should be identified using tractography methods, although this was beyond the scope of our study. Finally, our statistical approach followed the standard MRtrix3 FBA pipeline, which involves performing separate statistical tests for each fixel-based measure (FD, FC, and FDC). This method may increase the risk of false positives due to multiple comparisons. Using an F-test to simultaneously compare all three measures could offer stronger control of the family-wise error rate. Future studies should consider adopting multivariate methods and optimizing data acquisition parameters, while also accounting for sex differences in their analyses, to gain a more comprehensive understanding of the structural differences in older adults and the underlying mechanisms.

## Conclusions

In summary, our study provides additional insights into white matter differences between younger and older adults, utilizing a precise and interpretable approach known as fixel-based analysis. These findings underscore the complexity of age-related changes, involving both microstructural and macrostructural effects associated with variations in fiber density, cross-section, and FDC. Our study demonstrates that fixel-based analysis could be valuable for exploring the white matter correlates of cognitive decline in older individuals.

## CRediT authorship contribution statement

**Feliberto de la Cruz:** Conceptualization, Writing – review & editing, Writing – original draft, Methodology, Formal analysis. **Andy Schumann:** Writing – review & editing, Writing – original draft, Data curation, Conceptualization. **Katrin Rieger:** Writing – review & editing, Writing – original draft, Project administration, Data curation. **Daniel Güllmar:** Writing – review & editing, Writing – original draft, Methodology. **Jürgen R. Reichenbach:** Writing – review & editing, Writing – original draft, Methodology. **Karl-Jürgen Bär:** Writing – review & editing, Writing – original draft, Supervision, Investigation, Conceptualization.

## Declaration of competing interest

The authors declare that they have no known competing financial interests or personal relationships that could have appeared to influence the work reported in this paper.
